# Supplementary material for: Is scientific evidence enough? Using expert opinion to fill gaps in data in antimicrobial resistance research
Source: PLoS One. 2023 Aug 24;18(8):e0290464. doi: 10.1371/journal.pone.0290464 (PMC10449168; doi:10.1371/journal.pone.0290464)
Supplement: Attachment — Submitted filename: Response to Reviewer.docx [file pone.0290464.s001.docx]

Dear Dr. Dinga,

We thank you and the reviewer for your comments on manuscript PONE-D-23-05382 (Is scientific evidence enough? Using expert opinion to parameterize a simulation model of antimicrobial resistance in Sweden), that have helped us make the manuscript and objectives clearer, and better highlight the usefulness of the methods taken to identify novel forms of data to help further research in antimicrobial resistance (AMR).

We have addressed all reviewer comments in detail below. Line numbers below refer to the marked version of the revised manuscript.

Per your editorial comments, we have added details regarding participant consent, including the specified type of consent (written) that was collected and a full ethics statement, to the Methods Section (Lines 122-124) and online submission information.

Regarding any previous publication of the work, please note that the only previous version of this work is my PhD thesis. This document is housed in my institution’s repository (<http://hdl.handle.net/10012/18478>) and I own the full copyright to the thesis. This document was not peer-reviewed nor is it formally published. The content of this manuscript is a revised version of the previous thesis version that was revised by a wider range of authors, the country names were de-identified within the quotations by the experts, and the results were further synthesized and framed within the broader context of current AMR research.

We have ensured the manuscript meets PLOS ONE’s style requirements, including the reference style, title page format, sub-title formatting, and file names. Please note there are no supplementary materials to accompany this manuscript and therefore no captions are required. The supplementary materials that were referenced in the original manuscript were outlining where to locate specific figures within a different study (Lambraki et al., 2022). Therefore, to avoid confusion, these details have been removed and the references to the general article have remained.

Sincerely,

Melanie Cousins

**RESPONSE TO REVIEWER COMMENTS**

**Reviewer comment:**

*The abstract and introduction are slightly misleading since they use expressions such as “parametrizing a model” that give the impression that a mathematical model of AMR drivers was developed. In fact, the outcome of the study is simply a concept map from an expert workshop. This could be stated more explicitly.*

**Response:**

Thank you for this comment. We have revised the manuscript to outline our objectives more clearly with the results of the paper. Specifically, the title, abstract (Lines 4-10), rationale (Lines 67-80), and objectives (Lines 80-83) have been refined to better describe the intended goals of this manuscript. The title of the manuscript was updated to: “Is scientific evidence enough? Using expert opinion to fill gaps in data in antimicrobial resistance research” to better reflect the main research objective, which was to derive quantitative estimates from experts’ statements to begin to fill these data gaps.

Please note that the causal loop diagram (CLD; the concept map to which you refer) was created during the original workshop and described/published by Lambraki et al., 2022. Specifically, Lambraki et al produced a visual image of the drivers and their interconnections. This manuscript advances the work of Lambraki et al by identifying quantitative estimates for the various drivers and their associations (e.g., strengths and directions) and adding them to the Lambraki et al CLD. The major contribution of this work was not to identify new concepts or divers of AMR, but as an intermediate step to fill gaps for where quantitative data does not yet exist or is limited.

**Reviewer comment:**

*The number of study participants is also only 17 and highly under-representative, mostly consisting of AMR professionals/experts from Sweden.*

**Response:**

To address the concerns about the under-representativeness of the study, we have added descriptions about participant characteristics (Lines 163-175) and information within the limitation sections (Lines 597-602).

Overall, our participants had varying levels of expertise in AMR. About half were not experts in AMR, but rather were invited to participate because they possessed expertise in sectors or areas that may impact or be impacted by AMR.  This was important to ensure our participants brought expertise from multiple sectors including those not traditionally considered when assessing upstream drivers and downstream impacts, including how those factors influence other factors and how they increase/decrease when impacted or impacting other factors.

We set out to recruit 12 to 20 participants representing diverse perspectives and successfully recruited 17 participants across human and animal sectors. Smaller sample sizes are common in qualitative research because the focus is on selecting information rich cases to adequately explore a phenomenon of interest, like AMR, in greater depth (Sandelowsk, 1996; Patton 2002; Cresswell & Planko Clark, 2011). Lincoln and Guba (1985) proposed that sample size determination be guided by the criterion of informational redundancy, that is, sampling stops when no new information is elicited. To ensure informational redundancy (saturation of issues discussed) our workshops were designed to provide participants with sufficient time to describe the factors/drivers and values that may inform the development of a quantitative mode, ensured participants had time to question, contest, and build on each other’s comments (facilitation techniques) and kept questioning and probing participants until we heard no new information emerge and participants themselves indicated to us they had nothing new to share. Moreover, our two workshop sessions each had different people participate (7 on day 1; 10 on day 2). Issues raised during the two sessions were similar, providing another indication of information redundancy. In addition, participatory systems dynamics modelling recognizes that while no systems model is 100% correct, because the external landscape is ever-changing, the CLDs and what participants described reflect the best estimation of the dynamics that influence AMR in the European context based on the perspectives of the participants at the time of data collection (Hummelbrunner, 2009). Thus, our approach to sampling followed accepted approaches to sampling in qualitative research and in establishing saturation of core issues/themes. Finally, this study provided semi-quantitative data for 83 nodes and various relationships and highlights that such data can be derived from this type of expert engagement. Because this study showed that useful information can be derived through these qualitative methods, future studies should expand participant engagement, including to those from sectors not at our workshop (e.g., environment) to populate the nodes and relationships of the CLD.

**Reviewer comment:**

*Moreover, there was only 61 % consistency in the coding when three independent researchers coded the data. Even though the definition of the categories of the codes was refined and this was stated to result in consensus among the reviewers, it appears that intercoder reliability was not reassessed. Therefore, there may be considerable reliability issues caused by the subjective nature of the coding.*

**Response:**

Thank you for this comment. We would like to clarify that there was only 61% consistency between the reviewers in the original test coding. At this stage, the inconsistency came from one coder and the contradictions were minor, with most discrepancies due to the coder’s misunderstanding on the different sources of knowledge (mainly personal versus professional). We did not re-quantify intercoder reliability because the pre-agreed intent of our consensus-based approach to intercoder reliability was focused on fostering dialogue and reflexivity and surfacing discrepancies in coding to clarify, discuss and come to agreement on how to resolve the conflicting interpretations responsible, and using that information to refine the coding frame (e.g., better define codes that may be ambiguous) than a focus on a final score; reaching 100% consensus about our discrepancies was our indicator that satisfactory reliability had been reached and is an approach that is commonly used by others who conduct intercoder reliability checks with or without generating a score (Barbour, 2001; O’Connor & Joffe, 2022; Joffe & Yardley, 2003; Campbell et al., 2013; Cheunga & Tai, 2021).

Details about the inter-coder reliability process has been added to the manuscript (Lines 252-258).

**Reviewer comment:**

*In summary, the study makes a relatively weak and minor contribution to the understanding of AMR drivers. Therefore, to justify its consideration as a novel and rigorously conducted study to be accepted for publication in a scientific journal, the manuscript should be edited to (more) clearly answer the following questions:

1) How is this study precisely distinct from and what is its novelty compared to Lambraki et al., 2022, that it is claimed to extend?* **Response:**

In response to the question posed, the following description was added to the methods section to explicitly outline how this study differs from the original workshop paper outlined by Lambraki et al., 2022:

“While the original study outlined in Lambraki et al., 2022 produced a CLD of the system that drives AMR and thematically described the major areas of the system and potential places to intervene, it did not quantify the current state of the factors or put strengths to the relationships between the factors. Therefore, the transcripts from the workshop were re-analyzed to begin to quantify the CLD by identifying quantifiable data from the experts’ statements for the factors and strengths and directions of the relationships between the factors.” (Lines 113-119)

*2) How do the study findings expand on and relate to previous scientific discussion and findings concerning the drivers of AMR? Most of the drivers identified do not seem novel to me, and the results and discussion do not clearly contextualize the study findings in terms of previous research on AMR drivers.*

**Response:**

As a response to the question above, the following clarifications were added to the Discussion section (Lines 636-663, 704-716).

In short, the aim of this study was not to identify drivers (as was done in Lambraki et al, 2022) but to provide quantitative estimates to the drivers and the relationships between the drivers which have not or are hard to be quantified. Many studies of the drivers of AMR outline the system qualitatively but do not take the next step to begin to get experts to quantify the drivers. Many models of AMR are limited in scope because the estimates for cross-sector impacts have not been quantified (e.g., animal-environment or environment-human). Source attribution and whole genome sequencing work has begun to better understand these connections, but the data is still limited. Using expert knowledge in the interim is a good solution.

**Reviewer comment:**

*In addition, another major point is that the authors should more transparently express that what they present in this study is (simply) a workshop concept map. The current use of elaborate language (e.g. model parametrization) can mislead readers regarding the methodology and importance of the study, considering it to be a mathematical modeling paper.*

**Response:**

Overall, we believe that the changes we have made above (reframing the title and objectives) have adequately addressed this concern. Furthermore, since we did not want to lose the concept of the future application of this method to further modelling of AMR, this has been touched on as a recommendation for future research in the Discussion section (Lines 701-704, 752-754).

**REFERENCES:**
Barbour, R. S. (2001). Checklists for improving rigour in qualitative research: A case of the tail wagging the dog? British Medical Journal, 322, 1115–1117. https://doi.org/10.1136/bmj.322.7294. 1115

Campbell, J. L., Quincy, C., Osserman, J., & Pedersen, O. K. (2013). Coding in-depth semistructured interviews: Problems of unitization and intercoder reliability and agreement. Sociological Methods & Research, 42, 294–320. https://doi.org/10.1177/0049124113 500475

Joffe, H., & Yardley, L. (2003). Content and thematic analysis. In D. F. Marks & L. Yardley (Eds.), Research methods for clinical and health psychology (pp. 56–68). Sage.

Ka Ching Cheunga and Tai (2021). The use of intercoder reliability in qualitative interview data analysis in science education. https://doi.org/10.1080/02635143.2021.1993179

Lambraki IA, Cousins M, Graells T, Leger A, Henriksson P, Harbarth S, et al. (2022). Factors influencing antimicrobial resistance in the European food system and potential leverage points for intervention: A participatory, One Health study. PLoS One 17:1–19. https://doi.org/10.1371/journal.pone.0263914.

Lincoln YS, Guba EG. Naturalistic inquiry. London: Sage; 1985.

O’Connor and Joffee 2020: International Journal of Qualitative Methods; Volume 19: 1–13 DOI: 10.1177/1609406919899220

Patton MQ. *Qualitative research and evaluation methods.* 3rd Sage Publications; Thousand Oaks, CA: 2002.

Sandelowski M. One is the liveliest number: the case orientation of qualitative research. Res Nurs Health. 1996;19(6):525–9.

Williams B, Hummelbrunner R. *Systems concepts in action. A practitioner's toolkit.* Stanford, California: Stanford University Press; 2009
